# IMPLEMENTING A NEW MODEL IN PRIMARY CARE FOR OLDER CANADIANS LIVING WITH FRAILTY

**DOI:** 10.1093/geroni/igz038.2537

**Published:** 2019-11-08

**Authors:** Jacobi B Elliott, Joanie Sims Gould, Susie Gregg, Catherine Tong, Paul Stolee

**Affiliations:** 1 University of Waterloo, Waterloo, Ontario, Canada; 2 University of British Columbia, Vancouver, British Columbia, Canada

## Abstract

Primary care may be the best place within the health system to coordinate care for older persons, but at present, it is poorly equipped to do so. Effective models for complex patients require appropriate targeting, patient/caregiver engagement, and care coordination. A large national project aims to co-design and implement a model in primary care that includes risk-stratification, patient engagement and care coordination techniques for older adults. This presentation focuses on the process of implementation in primary care. Grounded in the Consolidated Framework for Implementation Research, researchers worked with nine primary care sites in three Canadian provinces. Project implementation was completed in two phases. Pre-implementation: Interviews with providers (n=25) and older adults (n=8) were conducted to understand current practices and plan for implementation. Implementation: Researchers worked with sites to train staff and support implementation. Monitoring of the implementation process included Interviews with providers (n=20) and field notes. Data were analyzed using directed coding, following the framework. A number of learnings emerged: buy-in was required from the entire team, teams provided meaningful information to guide implementation, contributing to a sense of ownership, and it was important that intervention components were tailored to the needs at each site. Ongoing and frequent discussions with the team was necessary. Scheduling meetings and training sessions for providers was challenging due to the length of time away from direct patient care. A new primary care model for older adults living with frailty was implemented. Lessons from this project will be used to guide future implementation and spread.

